# Pretransplant Levels of CRP and Interleukin-6 Family Cytokines; Effects on Outcome after Allogeneic Stem Cell Transplantation

**DOI:** 10.3390/ijms17111823

**Published:** 2016-11-01

**Authors:** Tor Henrik Tvedt, Stein Atle Lie, Håkon Reikvam, Kristin Paulsen Rye, Roald Lindås, Tobias Gedde-Dahl, Aymen Bushra Ahmed, Øystein Bruserud

**Affiliations:** 1 Section for Hematology, Department of Medicine, Haukeland University Hospital, 5021 Bergen, Norway; Hakon.Reikvam@uib.no (H.R.); roald.lindas@helse-bergen.no (R.L.); aymen.bushra.ahmed@helse-bergen.no (A.B.A.); oystein.bruserud@helse-bergen.no (Ø.B.); 2 Department of Clinical Dentistry, University of Bergen, 5020 Bergen, Norway; Stein.Lie@uib.no; 3 Section for Hematology, Institute of Clinical Science, University of Bergen, 5020 Bergen, Norway; Kristin.Rye@uib.no; 4 Department of Hematology, University of Oslo, 0424 Oslo, Norway; tgeddeda@ous-hf.no

**Keywords:** allogeneic stem cell transplantation, interleukin 6, interleukin 31, C reactive protein, graft versus host disease, comorbidity, fluid retention

## Abstract

Several pretransplant factors, including CRP (C-reactive protein) levels, reflect the risk of complications after allogeneic stem cell transplantation. IL-6 induces CRP increase, and we therefore investigated the effects of pretransplant IL-6, soluble IL-6 receptors, IL-6 family cytokines and CRP serum levels on outcome for 100 consecutive allotransplant recipients. All patients had related donors, none had active infections and 99 patients were in complete remission before conditioning. The incidence of acute graft versus host disease (aGVHD) requiring treatment was 40%, survival at Day +100 82%, and overall survival 48%. Despite a significant correlation between pretransplant CRP and IL-6 levels, only CRP levels significantly influenced transplant-related mortality (TRM). However, CRP did not influence overall survival (OS). Pretransplant IL-31 influenced late TRM. Finally, there was a significant association between pretransplant IL-6 and early postconditioning weight gain (i.e., fluid retention), and this fluid retention was a risk factor for aGVHD, TRM and OS. To conclude, pretransplant CRP, IL-31 and early posttransplant fluid retention were independent risk factors for TRM and survival after allotransplantation.

## 1. Introduction

Graft versus host disease (GVHD) and severe infections are the most important causes of non-relapse mortality after allogeneic stem cell transplantation (ASCT) [1,2]. The risk of GVHD is influenced by pre-existing patient-, donor- and disease-specific factors as well as the pretransplant conditioning treatment and GVHD prophylaxis. The pretransplant cytokine network is also important, and experimental models suggest that the conditioning therapy induces the release of pro-inflammatory cytokines that increase the MHC (Major histocompatibility complex) molecule expression on host antigen-presenting cells and thereby activates donor T cells [3]. Several studies also suggest that specific single nucleotide polymorphisms (SNP) in Interleukin-6 (IL-6) genes influence the risk and severity of acute GVHD [4].

Previous analyses have shown that pre-transplant CRP levels correlate with overall survival (OS) and transplant-related mortality (TRM) [5,6,7,8,9,10,11]. The molecular mechanisms behind these associations are largely unknown and only one study included analysis of cytokines together with CRP [5]. IL-6 is produced by macrophages and mesenchymal cells during inflammation and is the main driver of CRP production. The IL-6 cytokine family includes IL-6 together with IL-11, IL-27, IL-31, Leukemia inhibitory factor (LIF), Oncostatin M (OSM), Ciliary neutrophilic factor (CNTF), Cardiotrophin-1, Cardiotrophin-like-cytokine and Neuropoietin [12]. All these cytokines bind to receptors utilizing gp130 for signal transduction and are involved in immunoregulation [13,14,15]. Mice depleted of IL-6 still retain their ability to produce CRP [16]. Experimental studies suggest that the other IL-6 family members then compensate for the IL-6 response by interacting with IL-6R and causing an acute phase reaction. Cross-reactivity between other IL-6 family cytokine receptors is also possible [15,17].

Animal studies suggest that IL-6 is important in GVHD pathogenesis and inhibits reconstitution of regulatory T-cells, thereby promoting Th17 development [18,19,20,21]. However, IL-6 is also linked to anti-inflammatory processes and tissue regeneration [22]. The IL-6 receptor lacks intracellular domains and relies on gp130 for intracellular signal transduction. gp130 is ubiquitously expressed, whereas the membrane-bound IL-6 receptor (IL-6R, also known as CD126) is only found on certain cells. Soluble IL-6R (sIL-6R) does not inactivate IL-6, but binds to and activates gp130 on cells not expressing IL-6R themselves. Activation by sIL-6R is thought to mediate mainly pro-inflammatory effects while activation through membrane-bound IL-6R mainly mediates anti-inflammatory effects.

Under physiological conditions, soluble gp130 (sgp130) levels exceed the sIL-6R levels and thereby act as a physiological buffer against pro-inflammatory IL-6 effects [23]. Specific SNPs in IL-6R lead to higher levels of sIL-6R and are also associated with higher baseline CRP [24,25] and increased incidences of inflammatory and cardiovascular diseases [26]. IL-6R levels are also associated with increased relapse rate in certain cancers [27].

As described above, the IL-6 family cytokines have important immunoregulatory functions, but they also function as regulators of vascular permeability [28,29,30,31]. In this context, we have investigated the possible associations between pretransplant levels of CRP/IL-6 family members and posttransplant outcomes, including early weight gain (i.e., fluid retention) as well as GVHD and survival.

## 2. Results

### 2.1. The Clinical Characteristics of Patients Included in the Study

During the observation period a total 102 ASCTs were performed, including one ALL (acute lymphoblastic leukemia) and one AML (acute myeloid leukemia) patient who were re-transplanted due to relapse. The characteristics of the 100 patients are summarized in Table 1; 95 of these patients were Caucasians. Pretransplant serum samples were available for 100 transplantations (i.e., 98 patients included in the study). The median time from samples collection until transplantation was 23 days (interquartile range (IQR) 14 days), and average storage time before analysis 1518 days (range 75–3464 days, IQR 1688 days).

At admission for transplantation, 95 patients had Performance Status (PS) 0–1, only one patient had PS 3 due to immobilization secondary to prior cerebrovascular disorder caused by polycythemia vera, and no patients had PS 4. No patient had active infection and all but one AML patient were in remission when conditioning therapy started. With the exception of one patient, GVHD prophylaxis with cyclosporine A plus four doses of methotrexate (Days 1, 3, 6 and 11) was planned, but two of them did not receive methotrexate due to early complications. Antithymocyte globulin (ATG) was given to two patients as additional GVHD prophylaxis due to one HLA-antigen mismatch. All patients received granulocyte colony-stimulating factor (G-CSF) mobilized peripheral blood stem cell grafts except for patients with aplastic anemia (*n* = 4) or a donor younger than 15 years of age (*n* = 1) who all received bone marrow grafts. The majority of patients received conditioning treatment with BuCy (74 patients; busulfan 0.80 mg/kg QID from Day −7 to −5 and cyclophosphamide 60 mg/kg QD on Day −3 and −2) or FluBu (17 patients; fludarabine 30 mg/m^2^ QD from Day −9 until −5 and busulfan 3.2 mg/kg QD on Day −3 and −2) (Table S1); busulfan was always given intravenously. Sinusoidal obstruction syndrome was diagnosed according to the Baltimore criteria for six patients.

### 2.2. Pre-Transplant IL-6 and sgp130 Serum Levels Were Increased Prior to Conditioning Therapy Whereas the Levels of sIL-6R and Other IL-6 Family Members Did Not Differ from Healthy Controls

LIF serum levels were only analyzed for 34 unselected patients and five controls and could not be detected for any of them; to save sample material, analysis of LIF was omitted for the remaining patients. IL-11 and IL-28 serum levels were determined for all patients and controls, but since the majority of patients showed undetectable levels or levels close to the detection limit, both these mediators were excluded from the statistical analyses. The serum levels of the other mediators were included in our statistical analyses together with a new parameter referred to as IL-6 difference and defined as the serum level of sgp130 minus the corresponding level of sIL-6R.

Median serum level, variation range and IQR of each mediator for the patients and healthy controls are presented in Table 2 and Figure S1. IL-6 showed significantly higher levels for the patients compared to the healthy controls (*p*-value < 0.01); sgp130 levels were also higher in the patients but this difference reached only borderline significance (*p*-value 0.049), whereas sIL-6R levels did not differ significantly. The other IL-6 family members did not show any statistically significant differences when comparing patients and healthy controls.

The correlations between the levels of various IL-6 family members are presented in Table S1. A strong correlation was only seen between IL-6R and sgp130; in addition IL-6 showed significant correlations with both IL-6R and sgp130, whereas sgp130 also showed significant positive correlations with IL-6R and CNTF and an inverse correlation with IL-31.

### 2.3. Preconditioning Levels of IL-6 Family Cytokines Did Not Differ between Patients with and without Later aGVHD

Patients receiving RIC transplantations were significantly older, showed lower IL-31 levels but higher levels of sIL-6R and sgp130 than the MAC patients (Table S2); in both groups there were no correlation between age and mediator levels. Furthermore, there was no difference in mediator (IL-6 family members, soluble receptor chains) levels between patients experiencing later aGVHD and patients not developing aGVHD (see Table S3), but a non-significant trend of higher IL-31 levels was observed for patients with aGVHD (*p*-value 0.097).

### 2.4. sIL-6R and sgp130 Levels Correlates with Time until Neutrophil Reconstitution but Not with Time Until Platelet Reconstitution

Several IL-6 family members regulate normal hematopoiesis [32,33,34,35], and we therefore investigated whether their preconditioning systemic levels showed any correlations with preconditioning peripheral blood cell counts or posttransplant engraftment (see Table 3 and the complete data presented in Table S4). Firstly, IL-6 levels correlated inversely to pretransplant hemoglobin concentration (Table S4; Spearman’s ρ = −0.40, *p*-value < 0.05); this is similar to previous observations [36]. Secondly, OSM serum levels showed a statistically significant correlation with preconditioning total peripheral blood leukocyte counts (Spearman’s ρ = 0.27, *p*-value < 0.05), but without significant correlations to lymphocyte, neutrophil or monocyte counts. For the other IL-6 cytokine family members, no significant correlations were detected.

Preconditioning serum sIL-6R and gp130 levels showed significant positive correlations to time until neutrophil engraftment (Table 3). Furthermore, for patients with CRP level above median we observed a significantly lower pretransplant hemoglobin concentration, IL-6 concentration and leucocyte count; there was also a significantly higher proportion of CMV positive patients in the high CRP group (60.3% vs. 91.0%, *p*-value < 0.01).

### 2.5. Pretransplant IL-6 Levels Correlated with Pretransplant CRP Levels

The lower limit of detection for CRP was 1 mg/L. Median pretransplant CRP serum level was 5 mg/L (IQR 12 mg/L, range LLOD-120 mg/L). CRP correlated significantly to pretransplant IL-6 levels (Spearman’s ρ = 0.68, *p*-value < 0.05) and also to pretransplant hemoglobin level (Spearman’s ρ = −0.36, *p*-value < 0.05). No significant correlation between age and CRP levels was observed. There was no difference between median CRP levels for patients receiving RIC and MAC treatment (*p*-value 0.896).

### 2.6. A Large Patient Subset Shows Early Weight Gain after Conditioning/Transplantation

IL-6 seems to contribute to the increased vascular permeability during inflammation; similar effects have also been suggested for IL-11, IL-21 and possibly LIF [28,29,30,31]. For this reason, we investigated both the possible associations between pretransplant levels of IL-6 family members and posttransplant weight increase/fluid retention, and the impact of weight gain on outcome after transplantation. We first analyzed maximal weight gain by comparing contrasting groups. The median value of the maximal weight gain during the first four weeks after the start of conditioning therapy was 5.0 kg (range 0–16.1 kg, IQR 4.0 kg) weight increase compared with baseline. Only one of the patients with weight gain exceeding 5 kg was diagnosed with sinusoidal obstruction syndrome. Both pretransplant CRP (Generalized linear model as described in Material and Methods, *p* < 0.02) and IL-6 (*p* < 0.0) levels showed a significant effect on maximal weigh gain in univariate analyses, but in multivariate analysis no single factor had a significant effect on the weight gain.

The 50 patients with a maximal weight gain exceeding 5 kg could be divided into two groups depending on the time until maximum weight gain was registered; one group (21 patients) had a maximum weight gain prior to stem cell transplantation/infusion, and another group (29 patients) with increasing weight during the first two weeks posttransplant (Figure S2). We then analyzed the data for the whole patient population (*n* = 100); the median weight gain was then significantly higher for patients showing maximal weight after the transplantation compared with patients reaching their maximal weight gain between initiation of conditioning and stem cell transplantation (3.9 kg vs. 5.9 kg, *p*-value < 0.01), but the proportions of patients dying before Day +100 did not differ between these two groups (*p*-value 0.22). In this context, it was not unexpected that the time from start of conditioning treatment until maximum weight and maximal weight gain showed a significant correlation (Spearman’s test, ρ = 0.62, *p*-value < 0.01, Figure S2). There were no statistically significant differences in serum levels for any cytokine/mediator, CRP level, age or any other clinical/laboratory parameter when comparing patients with maximum weight gain above or below 5 kg.

**Figure 1:**
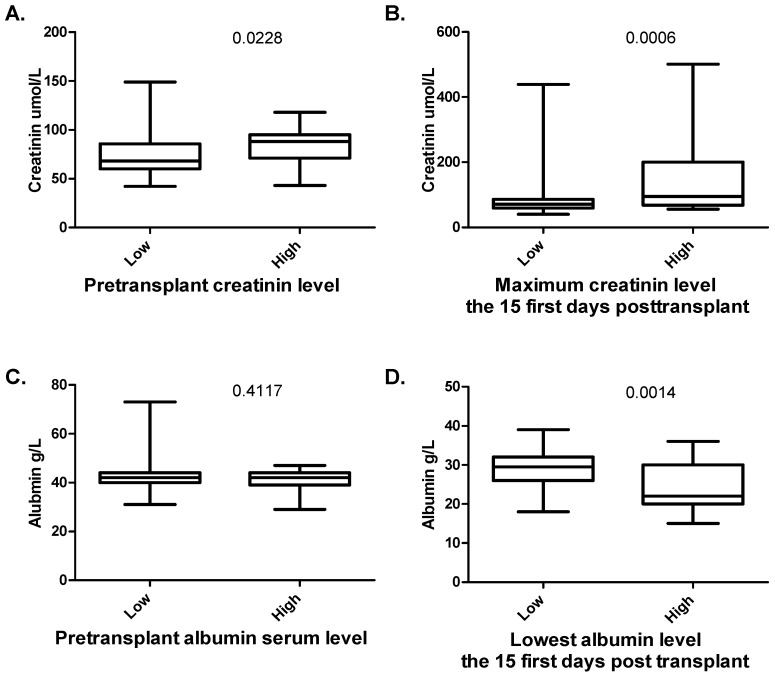
Maximal weight gain (i.e., fluid retention) early after allogeneic stem cell transplantation—a comparison between patients with weight gain below (**low**) or above (**high**) 5 kg. The figure shows the comparison of: (**A**) pretransplant creatinine serum level; (**B**) the highest observed creatinine level before Day +15 posttransplant; (**C**) pretransplant albumin levels; and (**D**) the lowest albumin level before Day +15 posttransplant. The Mann–Whitney *U*-test was used for the analyses; the corresponding *p*-value is given in the upper right for each part of the figure.

We then compared contrasting groups with high and low weight gain; based on the later use of maximal weight gain as a continuous variable and the use of dummy variables to define cut-off in the survival analyses (see section 2.8 below) we then used a cutoff of 6.8 kg to define two contrasting groups, i.e., patients with low and high weight gain, respectively. Firstly, the median pretransplant creatinine kevel for all 100 patients was 72 µM (variation range 42–149 µM). Patients with weight increase exceeding 6.8 kg had a significantly higher creatinine levels prior to conditioning therapy compared with the other patients (*p* = 0.02), and this differences remained significant also when comparing creatinine levels 14 and 28 days after transplantation. Secondly, the preconditioning albumin levels did not differ between these two groups, whereas the albumin levels were significantly lower for patients with maximal weight gain exceeding 6.8 kg both when comparing these two groups 14 and 28 days after transplantation (Figure 1). Thirdly, the cyclosporine A levels did not differ between the two groups.

### 2.7. The Risk of Steroid-Requiring aGVHD Was Only Associated with Maximum Weight Gain and Sibling vs. Non-Sibling Donor but Not with Preconditioning Levels of Cytokines/Receptors or CRP

The cumulative incidence of aGVHD requiring high-dose steroid treatment was 40%; this included patients with grade II disease with gastrointestinal involvement, and patients with grade III/IV acute GVHD. Only maximum weight gain and sibling vs. non-sibling donor were significantly associated with increased incidences of aGVHD, whereas we could not detect a significant effect for the preconditioning serum levels of any single mediator (IL-6 family cytokines, sIL-6R, and sgp130), CRP level, CMV status, female to male donor or age. The overall results of these univariate analyses are presented in Table S5, while the results from the multivariate analysis are presented in Table S4.

### 2.8. Transplant-Related Mortality before Day +100 Post-Transplant Was Only Associated with Maximum Weight Gain as Well as Preconditioning CRP and IL-31 Levels in Adjusted/Multivariate Analysis

**Figure 2:**
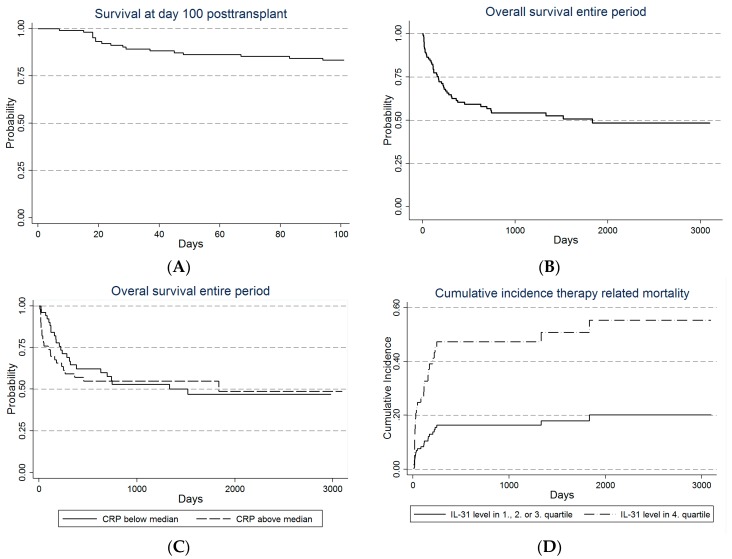
Survival after allogeneic stem cell transplantation for all patients included in our study. The Kaplan–Meier plots show: (**A**) overall survival for the first 100 days posttransplant and (**B**) overall survival for the entire period; (**C**) the effect on the overall survival of pretransplant CRP levels above or below the median CRP serum level; and (**D**) the cumulative incidence of TRM for patients with either low (quartiles 1–3) or high pretransplant IL-31 levels.

The Kaplan–Meier plot of overall survival 100 days post-transplant is shown in Figure 2A; the overall survival at Day +100 being 82% The crude analysis of TRM at Day +100 post-transplant showed significant associations with maximum weight gain, pretransplant IL-6 and IL-31 levels, type of transplantation and pretransplant CRP level above median (Table S6; *p*-values < 0.05). The effect of CRP on overall survival is also presented in Figure 2.

By splitting continuous variables into dummy variables it could be shown that possible cut-off points for IL-6 and IL-31 pretransplant levels corresponded to serum levels above the third quartile, for CRP above 14 mg/L and for maximum weight gain 6.8 kg. An adjusted model then showed a significant effect only of maximum weight gain (above vs. below 6.1 kg), pretransplant IL-31 serum level in the fourth quartile and pretransplant CRP levels above the median (Table S4). Dichotomizing variables were not regarded as optimal due to few events in each group, but an adjusted model with continuous variables showed significant effect of IL-31, CRP, maximum weigh gain and female to male transplantation.

### 2.9. Recipient Age, Maximum Weight Gain and Preconditioning IL-31 Levels Are Associated with Transplant-Related Mortality and Overall Survival after 2 Years in Multivariate Analysis

OS for the entire cohort at two years was 56%. In univariate analysis maximum weight gain, pretransplant CRP, pretransplant IL-31 serum level and sibling vs. non-sibling donor had significant effects on transplant-related mortality (Table S7). Pretransplant CNTF serum level was also included in the following multivariate analysis because it showed a *p*-value of borderline significance (*p*-value 0.08). In this final model age, maximum weight gain, pretransplant CRP level and pretransplant IL-31 serum level had significant effects on TRM (Table S8). However, only age, maximum weight gain and IL-31 level affected overall survival in uni- and multivariate analysis with no significant effect of CRP level.

### 2.10. Only Maximum Weight Gain and Preconditioning Serum IL-31 Levels Are Associated with Transplant-Related Mortality and Overall Survival for the Entire Observation Period in Multivariate Analysis

The median observation period of all patients was 477 days (range 7–3098 days). OS for the entire cohort is shown in Figure 2B. In univariate analysis, TRM was significantly influenced by maximum weigh gain, pretransplant CRP, pretransplant IL-31 level and sibling vs. non-sibling donor (Table S9), but in the final model only the effects of weight gain and IL-31 reached significance with no effect of CRP and sibling vs. non-sibling donor. Analysis for OS yielded similar results (Table S10). The effect of CRP on overall survival and IL-31 on TRM are shown in Figure 2C,D.

## 3. Discussion

Several studies have investigated the pro-inflammatory cytokine network after allogeneic stem cell transplantation. However, relatively few studies have investigated the impact of inflammation and cytokine levels prior to the conditioning therapy, but they suggest that preconditioning signs of inflammation (i.e., CRP levels) are important for the posttransplant clinical course (Table S11). The molecular mechanisms behind this prognostic impact of CRP are largely unknown. The systemic pretransplant cytokine profile, β_2_-Mikroglobulin serum levels and levels of endothelial cell markers also seem to reflect the risk of severe posttransplant complications [37,38]. IL-6 is the main driver of CRP production [39,40], and to further characterize the molecular mechanisms behind the pretransplant pro-inflammatory phenotype we investigated whether systemic preconditioning levels of IL-6 family members reflect the risk of posttransplant complications.

Our patient cohort is relatively small, but we would emphasize that our patient cohort represents an unselected and population-based group of patients, and the patient characteristics are in addition described in detail. Our cohort should therefore be regarded as representative for adults transplanted with HLA-matched family donor allografts. Although our patients represent an unselected consecutive cohort, the patient heterogeneity is relatively small compared with many other studies. Only family donors (and for almost all patients sibling donors) were used, nearly all patients received peripheral blood mobilized stem cells and most patients received the same conditioning treatment and GVHD prophylaxis. However, we would emphasize that our results have to be interpreted with care due to the relatively low number of patients and the patient heterogeneity, and future studies have to clarify whether these mechanisms are important also for other allotransplant recipients.

IL-6 can be constitutively released by and also be a growth factor for malignant hematopoietic cells [41,42], and high levels may even reflect an adverse prognosis in various malignancies [43,44,45]. IL-6 is also an important immunoregulator and sgp130 as well as sIL-6R influence both IL-6 and CRP levels. For these reasons we investigated whether systemic levels of IL-6, other IL-6 family members or sgp130/sIL-6R reflect a risk of posttransplant complications or disease relapse in allotransplant recipients.

Most previous studies have found the preconditioning CRP level to be an independent prognostic factor associated with increased TRM and subsequently OS; two studies also identified increased CRP levels as a risk factor for later aGVDH but only one study identified high CRP levels as a risk factor for cGVHD (Table S11). Disease status can influence pre-transplant CRP levels, and even though classification of disease status was not clearly defined or differed between these studies, it seems clear that all these previous studies included a relatively high number of patients with active disease. In addition, patient and donor heterogeneity together with several outcome possibilities makes it hard to draw robust conclusions from these studies. Our current study differs from previous studies in that the patient population is more homogeneous with respect of donor type, pretransplant disease status, performance status and conditioning regimens. In our study pretransplant CRP showed a strong association with TRM at Day +100, but this effect was lost over time with no significant effect on overall survival for the whole observation period. Thus, our results suggest that preconditioning CRP is an independent marker for risk of early death in allotransplant recipients with low disease burden.

In our present study, we included the early posttransplant weight gain in our statistical analyses together with preconditioning/pretransplant levels of IL-6 family cytokines. Endothelial cells express gp130 but not membrane-bound IL-6R. During inflammation increased IL-6 and sIL-6 levels cause activation of vascular endothelial gp130 leading to redistribution of VE-cadherin with disruption of adherence junctions between endothelial cells and subsequent capillary leakage [28]. Other members of the IL-6 family also play a role in the regulation of vascular permeability [28,29,30,31].

Very few studies have investigated early posttransplant fluid retention as a risk factor after allotransplantation. A weight increase of at least 3% during 24 h is often used as a part of the diagnostic criteria for capillary leak syndrome [46]. This definition was used in a recent study of capillary leak syndrome in elderly allotransplanted pediatric patients whereas a weight criteria alone was used for the smallest children; these authors then described an association between capillary leak syndrome and decreased survival [47]. In our present study we used a maximal increase of 5 kg in the body weight despite diuretic therapy as a cutoff for comparison of contrasting groups with regard to the degree of fluid retention. Furthermore, our previous studies suggest that this cut-off identifies two patient subsets that differ with regard to metabolic regulation of fluid balance and capillary permeability, i.e., altered levels of metabolites involved in regulation of vascular functions, endothelial function/damage, capillary permeability and renal functions [47]. Weight gain should thus be regarded as a posttransplant parameter influenced by the pretransplant status [48]. For these reasons, early posttransplant weight gain was included in our statistical analyses together with other preconditioning factors. Our studies showed that this early posttransplant weight gain was associated with adverse prognosis, but further studies are needed to clarify the biological mechanisms behind these associations.

There is no generally accepted definition for capillary leak syndrome [49], but a definition including at least 3% weight gain during 24 h may be used [46]. As an alternative we therefore analyzed the impact of the maximal weight gain, and in contrast to the definition of capillary leak syndrome our parameter could be handled as a continuous variable in the survival analyses. A high posttransplant weight gain was associated with high preconditioning creatinine level, decreased albumin levels at the time of maximal weight and increased aGVHD/transplant-related mortality later posttransplant. However, both maximal weight gain and the alternative definition of capillary leak syndrome seem to reflect complications that usually develop during the early posttransplant period before Day +15, suggesting that these two parameters at least partly reflect the impact of the same biological mechanisms.

Only Artz et al. [5] incorporated cytokine levels (IL-6) in their analysis of preconditioning CRP levels, and they could not detect any association between IL-6 above the median level and infections or hepatic toxicity (grade 3/4 at Day +100), duration of hospital stay, aGVHD, TRM or OS. By dichotomizing the IL-6 in the initial analysis one can easily loose the effect of IL-6, and for this reason we deliberately did not choose to dichotomize continuous variables prior to the first univariate analysis. By applying this approach it was possible to identify new cut-off points. Our observations of significant associations with IL-6 levels in univariate analyses suggest that this parameter is a part of a more complex increased-risk pretransplant phenotype, although it cannot be used in the pretransplant risk evaluation.

CRP is not only a biomarker of inflammation, it seems to be an important component of the innate host defense and its monomeric form activates and induces pro-inflammatory cytokine release by endothelial cells [50,51]. The preconditioning CRP levels probably reflect a pro-inflammatory phenotype, but it is likely that the complete risk-associated phenotype is more complex involving different molecular mechanisms including immunoregulatory metabolites and cytokines (including IL-6 family members) as well as damaged or altered endothelial cells [47,48]. Taken together with our previous studies our present observations suggest that the preconditioning CRP levels function as a risk factor that integrates the pro-inflammatory effects of several pretransplant characteristics, including serum IL-6 levels that showed significant associations in univariate analyses, correlated with CRP levels and even may serve as a therapeutic target in aGVHD [52].

In this study, high IL-31 levels were associated with reduced overall long term survival without any association with aGVHD. To the best of our knowledge the role of IL-31 in allogeneic stem cell transplantation has not been investigated previously. Baseline patient characteristics and relapse rate did not differ between the low and high IL-31 groups. IL-31 is released during inflammation by different cell types, including keratinocytes, fibroblast and cells of the innate and adoptive immune system. The main role of IL-31 is in the interaction between epithelial surfaces (i.e., skin, lung, and gut) and the immune system [15]. Serum IL-31 levels correlate with disease activity for pruritic skin disorders, and IL-31 seems important in the pathogenesis of allergic asthma as well as ulcerative colitis and Crohn’s disease [53,54,55,56]. Increased IL-31 levels are also seen in non-Philadelphia chromosome myeloproliferative disorders [57]. A possible hypothesis is that increased preconditioning IL-31 levels reflect disturbed epithelial barriers (e.g., skin, airways, and gastrointestinal tract) that cause a long-lasting predisposition to inflammation and/or infection.

Our present study further emphasizes the importance of the precondition/pretransplant status of allotransplant recipients with regard to risk of posttransplant complications. The molecular mechanisms behind the adverse pretransplant pro-inflammatory phenotype are probably complex and largely unknown. Identification of CRP as a possible biomarker suggest that pro-inflammatory mechanisms are important, and the suggested link between pretransplant IL-6/inflammation/fluid retention/outcome suggests that altered endothelial function/vascular permeability are also involved.

## 4. Material and Methods

### 4.1. Patients

The study was approved by the local Ethics Committee (REK VEST 2013/ 634, Regional Ethics Committee III, University of Bergen, Bergen, Norway) and samples collected after written informed consent from patients at Haukeland University Hospital. In this period only patients with an available family donor was allotransplanted and therefore no transplantations with matched unrelated donors are included. These patients represent all allotransplanted adults from a defined geographic area (Norwegian Health Regions III, IV and V) with an available family donor. The decision to do an allotransplantation was taken by the Norwegian Advisory Board for Stem Cell Transplantation and based on national guidelines. Thus, our study should be population-based and include a random group of well-characterized patients. Samples were collected on the day of pre-transplantation evaluation or on the day of admission for stem cell transplantation.

Acute and chronic GVHD was diagnosed according to generally accepted criteria. All patients with aGVHD were evaluated using Glucksberg score, but patients who required more than 1 mg/kg/day methylprednisolone intravenous or an equivalent dose as GVHD treatment had grade II-IV aGVHD, i.e., patients with grade II disease and gastrointestinal involvement, and patients with grade III/IV acute GVHD. Neutrophil reconstitution was defined as three consecutive days with neutrophil counts of at least 0.2 × 10^9^/L, and platelet reconstitution as stable platelet counts exceeding 20 × 10^9^/L for at least 3 consecutive days without transfusions.

For a subset of patients in this cohort it has previously been shown that increased preconditioning/pretransplant levels of specific metabolites predicts capillary leak syndrome [47,58]. The maximum weigh gain was therefore included in the analysis. Weight at start of conditioning therapy was set as the reference weight, and the weight was thereafter registered prospectively twice daily until hematological reconstitution and thereafter every morning; the maximum weight gain during the first 30 days posttransplant was recorded. As used in previous studies, capillary leak syndrome was defined as a 5 kg weight gain from baseline despite diuretic therapy. The Baltimore criteria were used for diagnosis of sinusoidal obstruction syndrome; ultrasound examination was used when this diagnosis was suspected based on the clinical evaluation. The majority patients were treated with ursodeoxycholic acid from the start of conditioning therapy [59].

Performance status (PS) at time of admission for ASCT was recorded for every patient during the entire period. Standard comorbidity index scores (HCT-CI and EBMT-score) were not systematically implemented or register until after 2012 and were therefore available only for a minority of patients; for these reasons, it was only PS registered.

### 4.2. Healthy Controls

Control samples from healthy individuals were collected from 14 randomly chosen healthy blood donors at the local blood bank. No additional information about gender or laboratory values was registered.

### 4.3. Analysis of Soluble Mediator Levels in Serum Samples

Venous blood was collected onto sterile plastic tubes (BD Vacutainer^®^ SST™ Serum Separation Tubes, Becton-Dickenson; Franklin Lakes, NJ, USA) and allowed to coagulate for 120 min at room temperature before centrifugation (300× *g* for 10 min) and serum collection. Serum was immediately frozen and stored at −80 °C until analyzed. Repeated freezing and thawing were avoided. The samples were analyzed with Bio-Plex kits for IL-6, IL-11, IL-27(p28), sIL-6R (sCD126), LIF and IL-31 (Bio-Rad, Hercules, CA, USA), and Multiplex Assays (Millipore, Billerica, MA, USA) for CNTF and OSM. All samples were analyzed using Luminex^®^200™ Bio-Rad platform with program version 6.1 and all analyses were performed in duplicates strictly according to the manufacturer’s instructions. CRP was analyzed using an immunoturbidimetric method provided by Roche (Basel, Switzerland), and during the entire period the lower limit of detection for CRP was 1 mg/L.

### 4.4. Statistical Analyses

Statistical analyses were performed using the Statistical Package for the Social Sciences version 22.0 (IBM Corp.; Armonk, NY, USA), GraphPad Prism 5 (Graph Pad Software, Inc.; San Diego, CA, USA) and Stata Version 14 (StataCorp. 2009; Stata Statistical Software, College Station, TX, USA). Spearman’s correlation for bivariate samples was used for correlation analyses, the Mann–Whitney *U*-test was used to compare continuous variables and the Chi-Square tests or Fisher’s exact test were used to compare categorical variables. Differences were regarded as statistically significant when *p*-values < 0.05.

Overall survival was calculated using the Kaplan–Meier product limit method. The Cox proportional Hazzard model was used for calculating crude and adjusted hazard ratios (HR) for overall survival (OS). In a similar manner crude and adjusted subdistribution hazard ratios (SHR) were calculated using cumulative incidence regression methods as described in Fine and Gray [60] for therapy related mortality during the first 100 days post-transplant (defined as early TRM), 700 days post-transplant (defined as late TRM) and for the entire period. For competing risk analysis cause of death was either classified as relapse related or treatment related. In advance it had been defined that age, CRP and variables with *p*-value <0.1 in univariate analyses would be included in the final model for each defined time period. In the final model a *p*-value <0.05 was regarded as statistically significant. Generalized linear model was used to analyze the effect of different covariates on maximum weight gain.

For samples with a measured value below the lower level of detection (LLOD), the value was set to the LLOD in the statistical analyses. For the models of OS and TRM each variable was first entered as continuous variables. Variables with a significant effect were split into three dummy variables each corresponding to second, third and fourth quartile to examine if dichotomization was possible.

## 5. Conclusions

This study confirms that elevated CRP level above baseline increases the risk of early but not late death due to transplant related mortality, but it is not associated with an increased risk of GVHD. Pretransplant IL-6 levels are highly correlated with CRP levels but does not predict outcome after ASCT. IL-31 was the only member of the Interleukin-6 family that had an effect on outcome; in contrast to CRP IL-31 had a significant effect on long-term TRM. The occurrence of capillary leak syndrome was associated with both GVHD and a significant increase in transplant related mortality. The pretransplant pro-inflammatory phenotype is associated with an increased risk of sever posttransplant complications and is characterized by increased levels of CRP, IL-6 and sgp130 and suggests a possible link between pretransplant IL-6 and posttransplant capillary leak.

## Figures and Tables

**Table 1:** Clinical and laboratory characteristics of the 100 allotransplant recipients included in the study.

Age, Median and Range (Years)	47.5 (15–70)
Caucasian/non-Caucasian (number)	95/5
Diagnosis (number)	
AML	43
MDS-AML	16
Myelodysplastic syndrome (MDS), high-risk	4
Acute lymphoblastic leukemia	20
Chronic myeloid leukemia	2
Myelofibrosis	4
Chronic myelomonocytic leukemia	2
Myeloproliferative neoplasia, unspecified	2
Aplastic anemia	4
Chronic lymphocytic leukemia	2
Hodgkin’s lymphoma	1
Remission at transplantation (number)	99
aGVHD requiring high dose steroid treatment (number) ^1^	46
Conditioning regimes (number)	
Busulfan + cyclophosphamide (myeloablative condition)	74
Fludarabine + busulfan (reduced intensity conditioning)	17
Antithymocyte globulin + cyclophosphamide	4
Others	5
GVHD prophylaxis (number)	
Cyclosporine A + methotrexate	97
Cyclosporine A + mycophenolate mofetil	1
Cyclosporine A + methotrexate + antithymocyte globulin	2
Donor (number)	
Related	100
Sibling	93
Parent	6
Other related	1
Female/male donor	39/61
Female donor to male recipient	21
CMV pos. recipient	65
CMV pos. donor to neg. recipient	18
Stem cell source (number)	
Bone marrow grafts	5
G-CSF mobilized peripheral blood stem cell grafts	95
CRP mg/L (median and range; lower limit of detection being 1.0 mg/L)	5 (<1–120)
Maximum weight gain kg (median, range)	5.0 (0–16.1)

**^1^** The criteria for high-dose steroid treatment were acute GVHD grade II with gastrointestinal involvement or Grade III/IV acute GVHD.

**Table 2:** Pretransplant serum levels of IL-6 family cytokines for the allotransplanted patients (*n* = 100); a comparison with the levels for healthy individuals (*n* = 14). Significant values (*p* < 0.05) are highlighted in bold.

Mediator	All Allotransplant Patients			Healthy Controls			*p*-Value	LLOD
	Median	Range	IQR	Median	Range	IQR		
OSM	6.7	(6.7–89.3)	2.6	7.3	(6.7–111.9)	25.4	0.13	6.7
CNTF	701	(127–15,464)	1874	502	(127–11,819)		0.67	127
IL-6	12.6	(0.92–581)	19.6	3.0	(0.9–7.2)	4.2	**<0.01**	0.9
sIL-6R	11,580	(609–42,666)	10,722	8427	(4936–22,594)	10,541	0.09	18.7
sgp130	54,808	(8286–226,166)	60,005	39,776	(32,525–134,172)	67,302	**0.049**	81.0
sgp130-sIL-6R difference	4306	(−20,977–206,959)	48,710	32,283	(27,387–1,114,152)	58,499	0.10	NR
IL-31	7.12	(2.59–130.80)	7.52	8.70	(2.59–25.51)	8.62	0.1856	2.59

Abbreviations: Sgp16-sIL-6R diff, Difference between sgp130 and sIL-6R levels; IQR, Interquartile range; LLOD, Lower level of detection, NR, Not relevant.

**Table 3:** Correlation between preconditioning serum levels of soluble mediators and the peripheral blood cell counts tested before and following allotransplantation. The results are presented as the Spearman’s ρ and significant correlations (*p* < 0.05) are highlighted in bold. (**Upper part**) Significant correlations between IL-6 family cytokine levels tested before conditioning therapy and peripheral Boblood cell counts tested before immediately before initiation of conditioning treatment; (**Lower part**) Correlations between preconditioning serum mediator levels and peripheral blood cell counts (neutrophils and platelets) tested after allotransplantation. Time to neutrophil engraftment was defined as peripheral blood neutrophils above 0.2 × 10^9^/L on three consecutive days and time to platelet engraftment as peripheral blood thrombocytes above 20 × 10^9^/L on three consecutive days without platelet transfusions.

**Preconditioning Peripheral Blood Cell Counts**		
**Peripheral Blood Parameter**	**IL-6 Family Cytokine**	**Correlation**
Hemoglobin level	IL-6	**−0.40**
Total leukocyte count	OSM	**0.27**
**Hematopoietic Reconstitution after Allotransplantation**		
**Mediator**	**Neutrophils above 0.2 × 10^9^/L**	**Platelets above 20 × 10^9^/L**
IL-6	0.12	0.19
sIL-6R	**0.283**	0.12
sgp130	**0.238**	0.05
Diff	**0.215**	0.02
CNTF	0.19	0.01
OSM	0.01	−0.06

## Supplementary Materials

Supplementary materials can be found at www.mdpi.com/1422-0067/17/11/1823/s1.

## Abbreviations

aGVHD	Acute graft versus host disease
ALL	Acute lymphoblastic leukemia
AML	Acute myeloid leukemia
ASCT	Allogenic stem cell transplantation
BM	bone marrow
CD	Cluster of differentiation
cGVHD	Chronic graft versus host disease
CMV	Cytomegalovirus
CNTF	Ciliary neutrophilic factor
CRP	C-reactive protein
EBMT	European Society for Blood and Marrow Transplantation
G-CSF	granulocyte colony-stimulating factor
gp130	Glycoprotein 130
HCT-CI	Hematopoietic cell transplant comorbidity index
HLA	Human leucocyte antigen
IL	Interleukin
IL-6R	Interleukin-6 receptor
IQR	Interquartile range
LIF	Leukemia inhibitory factor
MAC	Myeloablativ conditioning
MDS	Myelodysplastic syndrome
MHC	Major histocompatibility complex
NR	Not reported
OS	Overall survival
OSM	Oncostatin M
PB	peripheral blood
PS	Performance status
RIC	Reduced intensity conditioning
sgp130	Soluble glycoprotein 130
sIL-6R	Soluble interleukin-6 receptor
SNP	Single nucleotide polymorphism
TRM	Transplant-related mortality
