# Supplementary material for: Pretransplant Levels of CRP and Interleukin-6 Family Cytokines; Effects on Outcome after Allogeneic Stem Cell Transplantation
Source: Int J Mol Sci. 2016 Nov 1;17(11):1823. doi: 10.3390/ijms17111823 (PMC5133824; doi:10.3390/ijms17111823)
Supplement: Supplementary file 1 [file ijms-17-01823-s001.pdf]

Supplementary Materials: Pretransplant Levels of CRP, Interleukin-6 Family Cytokines and Outcome after Allogeneic Stem Cell Transplantation

Tor Henrik Tvedt, Stein Atle Lie, Håkon Reikvam, Kristin Paulsen Rye, Roald Lindås, Tobias Gedde-Dahl, Aymen Bushra Ahmed and Øystein Bruserud

Table S1. Correlation between the preconditioning serum levels of IL-6 cytokine family members, IL-6R and sgp130. Spearman's rank order correlation test was used for the analyses. The results are presented as the Spearman's ϱ and significant correlations ($p < 0.05$) are highlighted in bold.

Parameter	IL-6R	sgp130	IL-31	OSM	CNTF
**IL-6**	**0.34**	**0.31**	0.17	0.028	−0.17
**IL-6R**		**0.71**	**−0.23**	0.03	0.18
**sgp130**			**−0.26**	0.11	**0.24**
**IL-31**				−0.06	−0.17
**OSM**					**0.16**

Table S2. Biological and clinical parameters of allotransplant recipients included in the study; a comparison of patients receiving reduced intensity conditioning (RIC, n = 17) and myeloablative conditioning (MAC, n = 83) treatment.

Parameter	RIC-Group		MAC-Group		*p*-Value
	Median	Range	Median	Range	
sIL6-R	18,022	4775–33,936	10,353	609–42,666	<0.01
sgp130	107,106	31,493–157,256	54,145	8286–226,166	<0.01
IL-31	LLOD	LLOD–25.5	7.0	LLOD–131	0.03
Gender	Female	5	Female	33	0.324
	Male	13	Male	49	
Acute leukemia (number)	AML	9	AML	50	0.1
	ALL	0	ALL	20	
Age (years)	61	22–70	43	15-62	<0.01
Time to neutrophil engraftment (days)	17	6 to 24	15	10 to 50	0.09

Table S3. Preconditioning serum levels of IL-6 family cytokines, sIL-6R sgp130 for the 100 allotransplanted patients; a comparison of patients with and without later aGVHD (all concentrations are given in pg/mL).

Parameter	No aGVHD		aGVHD		*p*-Value
	Median	Range	Median	Range	
IL-6	13.19	1.32–434.93	10.35	LLOD [1]–580.78	0.602
sIL-6R	10,520	4775–33,936	12,783	609.4–42,666	0.271
sgp130	54,158	31,493–170,849	55,209	8286–226,166	0.447
IL-6 difference [2]	43,502.5	25,002.5–145,868.00	46,843.35	−20,976.5–206,959	0.418
IL-31	7.28	LLOD–25.51	LLOD	LLOD–130.80	0.095
OSM	LLOD (6.68)	LLOD–86.14	LLOD	LLOD–89.29	0.530
CNTF	736	LLOD–15,464	577	LLOD–10,148	0.870

[1] LLOD, lower limit of detection; [2] The IL-6 difference was defined as the serum level of sgp130 minus the corresponding level of sIL-6R.

Table S4. Correlations between pretransplant levels of cytokines and receptors for IL-6 family cytokines, peripheral blood cell counts, serum levels of biochemical parameters and maximal weigh gain. The *p*-values were calculated using Spearman's rank order correlation. The results are presented as the Spearman's ϱ and significant correlations ($p < 0.05$) are highlighted in bold and underlined.

Parameter	IL-6	sIL-6R	sgp130	IL-6-diff	IL-31	OSM	CNTF
Hb	**−0.40**	−0.11	−0.17	0.17	−0.04	−0.12	0.01
Leukocytes	0.05	0.10	−0.01	−0.02	0.08	**0.27**	−0.02
Neutrophils	−0.01	−0.06	−0.10	−0.09	0.10	0.14	−0.03
Lymphocytes	−0.02	0.06	−0.02	−0.03	0.12	0.12	0.13
Monocytes	0.05	0.03	−0.13	−0.14	−0.01	0.01	−0.04
Thrombocytes	−0.25	−0.141	−0.18	−0.16	−0.17	−0.05	−0.15
CRP	**0.68**	0.14	0.07	0.04	0.13	0.15	−0.12
LDH	−0.08	0.17	0.07	0.04	−0.02	0.06	−0.11
Maximal weight gain	0.137	−0.03	0.05	0.05	−0.02	0.07	−0.04

Abbreviations: CRP, C reactive protein (mg/L); Hb, Hemoglobin concentration (g/100 mL); IL-6 diff, IL-6 difference; Lactate dehydrogenase count (U/L).

Table S5. Crude and adjusted subdistribution hazard ratios for aGVHD.

Covariate	Crude				Adjusted			
	p-Value	SHR	95% CI		*p*-Value	SHR	95% CI	
IL-6, continuous variable	0.79	1.00	0.99	1.01				
ILl-6R continues variable	0.31	1.00	1.00	1.00				
sgp130, continuous variable	0.51	1.00	0.99	1.00				
Diff, continuous variable	0.60	1.00	0.99	1.00				
IL-31, continues variable	0.43	1.01	0.98	1.03				
OSM	0.82	1.00	0.98	1.02				
CNTF continuous variable	0.37	1.00	1.00	1.00				
Age/10 year	0.33	1.10	0.89	1.37	0.15	1.16	0.95	1.41
Gender	0.15	1.52	0.86	2.70				
RIC vs. MAC	0.45	0.74	0.34	1.62				
Sibling vs. non-sibling	0.01	3.12	1.39	6.99	<0.01	3.76	1.87	7.54
Female to male vs. other	0.89	0.94	0.45	1.99				
CMV pos. donor to neg. recipient vs. other	0.66	0.82	0.35	1.95				
CRP, continuous variable	0.13	1.67	0.86	3.25				
CRP, value below vs. above median	0.68	1.00	0.98	1.01				
Maximum weigh gain, <6.8 kg vs. >6.8 kg	<0.01	1.14	1.04	1.25	<0.01	1.14	1.05	1.24

Table S6. Crude and adjusted subdistribution hazard ratios for treatment related mortality at 100 days post-transplant.

Covariate	Crude				Adjusted			
	p-Value	SHR	95% CI		p-Value	SHR	95% CI	
IL-6, continuous variable	0.04	1.00	1.00	1.01				
IL-6, all other values vs. value in 4. quartile	<0.01	4.01	1.48	10.93	0.12	2.43	0.78	7.51
IL-6R continues variable	0.49	1.00	0.99	1.01	0.08	2.98	0.85	10.33
s-gp130, continuous variable	0.92	1.00	0.99	1.01	0.02	4.79	1.29	17.67
Diff, continuous variable	0.78	1.00	0.99	1.01				
IL-31, continues variable	<0.01	1.02	1.01	1.02				
IL-31, all other values vs. value in 4. Quartile	0.02	3.43	1.24	9.47	0.01	3.78	0.85	10.33
OSM	0.10	0.93	0.87	1.02				
CNTF continuous variable	0.28	0.99	0.98	1.01				
Age/10 year	0.70	1.06	0.79	1.42	0.83	1.03	0.73	1.47
Gender	0.84	1.11	0.44	3.08				
RIC vs. MAC	0.25	0.30	0.04	2.28				
Sibling vs. non-sibling	0.29	2.10	0.53	8.47				
Female to male vs. other	0.18	2.06	0.71	5.99				
CMV pos. donor to neg. recipient vs. other	0.63	1.45	0.46	4.60				
CRP, continuous variable	<0.01	1.03	1.01	1.04				
CRP, value below vs. above median	0.02	4.59	1.32	15.94	0.04	3.83	1.01	13.75
Maximum weigh gain, <6.8 kg vs. >6.8 kg	<0.01	5.19	1.83	14.72	<0.01	6.18	2.23	17.15

Table S7. Crude and adjusted subdistribution hazard ratios for treatment related mortality at 700 days post-transplant.

Covariate	Crude				Adjusted			
	p-Value	SHR	95% CI		*p*-Value	SHR	95% CI	
IL-6, continuous variable	0.11	0.99	0.99	1.00				
IL-6, all other values vs. value in 4. quartile	0.21	1.00	0.99	1.01				
IL-6R continues variable	0.87	1.00	0.99	1.00				
sgp130, continuous variable	0.41	1.00	0.90	1.00				
Diff, continuous variable	0.35	1.00	0.99	1.00				
IL-31, continues variable	<0.01	1.02	1.01	1.02				
IL-31, all other values vs. value in 4. Quartile	0.01	2.87	1.29	6.40	<0.01	3.78	1.67	8.54
OSM	0.36	0.68	0.30	1.55				
CNTF continuous variable	0.11	2.80	0.99	1.01				
Age/10 year	0.12	1.02	0.99	1.04	0.05	1.27	0.99	1.63
Gender	0.68	0.81	0.29	2.26				
RIC vs. MAC	0.96	1.00	0.98	1.02				
Sibling vs. non-sibling	0.04	2.80	1.02	7.65	0.18	2.14	0.69	6.65
Female to male vs other	0.39	1.43	0.64	3.22				
CMV pos. donor to neg. recipient vs. other	0.97	1.02	0.37	2.83				
CRP, continuous variable	0.03	1.02	1.00	1.03				
CRP, value below vs above median	0.03	2.36	1.07	5.16	0.04	2.14	1.09	5.07
Maximum weigh gain, <6.8 kg vs. >6.8 kg	<0.01	3.35	1.50	7.50	<0.01	3.90	1.69	8.99

Table S8. Crude and adjusted subdistribution hazard ratios for overall survival at day 700.

Covariate	Crude				Adjusted			
	p-Value	SHR	95% CI		*p*-Value	SHR	95% CI	
IL-6, continuous variable	0.52	0.99	0.99	1.00				
IL-6, all other values vs. value in 4. quartile	0.59	1.00	0.99	1.01				
IL-6R continues variable	0.61	1.00	0.99	1.00				
sgp130, continuous variable	0.34	1.00	0.99	1.00				
Diff, continuous variable	0.33	1.00	0.99	1.00				
IL-31, continues variable	0.04	1.02	1.01	1.03				
IL-31, all other values vs. value in 4. quartile	0.04	2.03	1.02	4.06	<0.01	2.76	1.35	5.64
OSM	0.24	0.67	0.35	1.29				
CNTF continuous variable	0.26	1.80	0.64	5.06				
Age/10 year	0.02	1.03	1.00	1.05	<0.01	1.41	1.09	1.84
Gender	0.58	1.24	0.58	2.71				
RIC vs. MAC	0.98	0.99	0.98	1.02				
Sibling vs. non-sibling	0.02	1.32	1.03	1.69	0.34	1.81	0.52	6.20
Female to male vs. other	0.36	1.51	0.63	3.63				
CMV pos. donor to neg. recipient vs. other	0.47	0.73	0.30	1.74				
CRP, continuous variable	0.18	1.01	0.99	1.02				
CRP, value below vs. above median	0.43	1.27	0.70	2.34	0.48	1.26	0.66	2.39
Maximum weigh gain, <6.8 kg vs. >6.8 kg	0.01	2.28	1.89	4.89	0.01	2.30	1.17	4.53

Table S9. Crude and adjusted subdistribution hazard ratios for overall survival whole period.

Covariate	Crude				Adjusted			
	p-Value	SHR	95% CI		*p*-Value	SHR	95% CI	
IL6, continuous variable	0.20	1.00	1.00	1.01				
IL-6, all other values vs. value in 4. quartile								
il-6R continues variable	0.90	1.00	1.00	1.00				
sgp130, continuous variable	0.47	1.00	1.00	1.00				
Diff, continuous variable	0.42							
IL-31, continues variable	<0.01	1.02	1.01	1.02				
il31, all other values vs. value in 4. quartile	0.02	2.68	1.21	5.92	0.01	2.97	1.23	7.14
OSM	0.96	1.00	0.97	1.03				
CNTF continuous variable	0.08	0.99	1.00	1.00	0.22	1.00	1.00	1.00
Age/10 year	0.05	1.30	1.00	1.02	0.01	1.42	1.08	1.87
Gender	0.42	0.72	0.33	1.59				
RIC vs. MAC	0.65	0.79	0.29	2.18				
Sibling vs. non-sibling	0.06	2.68	0.97	7.44	0.09	2.66	0.85	8.31
Female to male vs. other	0.30	1.50	0.69	3.25				
CMV pos. donor to neg. recipient vs. other	0.23	1.54	0.72	3.76				
CRP, continuous variable	0.03	1.02	1.00	1.03				
CRP, value below vs. above median	0.03	2.32	1.09	4.91	0.10	2.08	0.88	4.95
Maximum weigh gain, <6.8 kg vs. >6.8 kg	<0.01	2.89	1.30	6.40	0.01	3.02	1.30	7.03

Table S10. Crude and adjusted subdistribution hazard ratios for TRM whole period.

Covariate	Crude				Adjusted			
	p-Value	SHR	95% CI		*p*-Value	SHR	95% CI	
IL-6, continuous variable	0.72							
IL-6R continues variable	0.689							
sgp130, continuous variable	0.392							
Diff, continuous variable	0.382							
IL-31, continues variable	0.03	1.02	1.01	1.03				
IL-31, all other values vs. value in 4. quartile	0.03	2.00	1.05	3.93	0.02	2.94	1.49	5.81
OSM	0.514							
CNTF continuous variable	0.972							
Age/10 year	0.01	1.39	1.1	1.76	<0.01	3.23	1.17	1.9
Gender	0.351							
RIC vs. MAC	0.228							
Sibling vs. non-sibling	0.368	1.32	1.03	1.69				
Female to male vs. other	0.164							
CMV pos. donor to neg. recipient vs. other	0.948							
CRP, continuous variable	0.305							
CRP, value below vs. above median	0.778				0.895	1.04	0.57	1.89
Maximum weigh gain, <6.8 kg vs. >6.8 kg	0.03	1.97	1.07	3.63	0.02	2.08	1.12	3.89

Table S11. The prognostic impact of pretransplant CRP levels in allotransplant recipients; a summary of previous studies investigating the effect of pretransplant CRP on outcomes after allogeneic stem cell transplantation.

Author	Year	Patient Number	Non-Malignant Disease	Related/Unrelated	Stem Cell Source		Remission/High Risk	HCT-CI	Effect on				
					BM	PBSC			aGVHD	OS	TRM	RRM	cGVHD
Artz [5]	2008	112 [a]	5	68/44	7	105	NR/52	Yes	Yes	Yes [b]	Yes	NR	NR
Pavlu [11]	2010	271	No, only CML	130/141	256	15	NR/113	Yes	NR	Yes	Yes	No	NR
Remberger [6]	2010	504	~16%	196/229	156	312	NR	NR	No	Yes	Yes [d]	Yes [e]	NR
Sakamoto [7]	2012	211	8/211	86/95	95	86	NR, 90/121	NR	No	No	Yes	No	Yes
Aki [9]	2012	106	No	97/9	0	106	22	Yes	NR	Yes	No	NR	NR
Sato [8]	2013	90	NR	39/51	58	24	NR/12%	NR	Yes	Yes	Yes	NR	NR
Jordan [10]	2013	349	11%	170/179	227	121	NR/113	NR	No	Yes	Yes	No [c]	NR

a: Data only for 81 patients; b: effect of CRP only shown in univariate analysis; c: Increased CRP at day of stem cell infusion showed significant higher relapsed related mortality; d: Only for the RIC cohort; e: Effect only in univariate analysis. Abbreviation: BM, Bone Marrow; G-CSF, Granulocyte colony-stimulating factor; NR, not reported; PBSC, peripheral blood stem cells, RRM Relapse-related mortality.

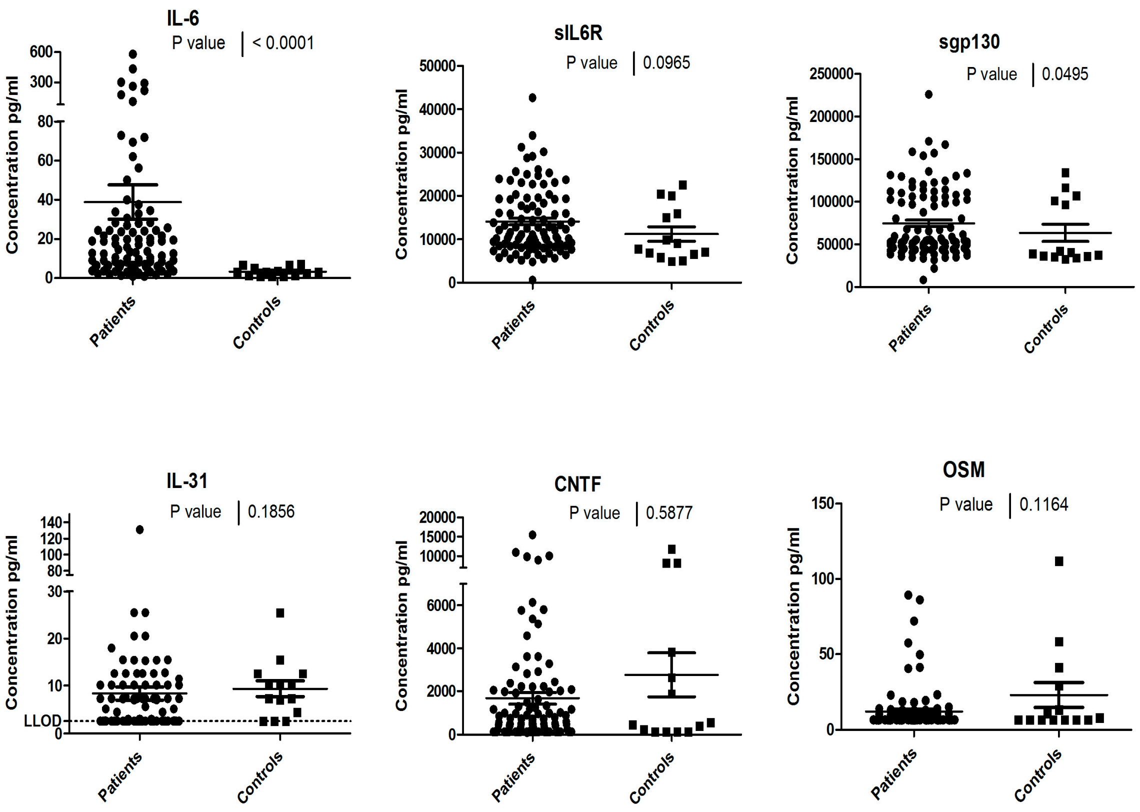

Figure S1. Preconditioning serum levels of IL-6 cytokine family members, IL-6R and sgp130; a comparison of between allotransplanted patients (n = 100) and healthy controls (n = 14) (LLOD = Lower limit of detection).

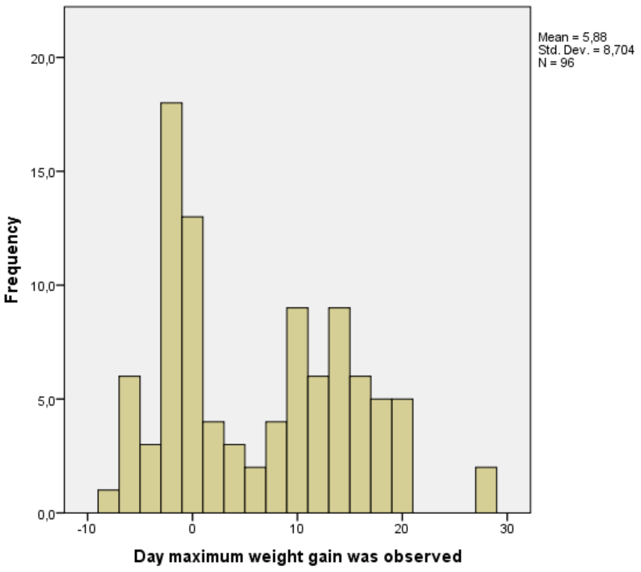

(**A**)

Figure S2. *Cont.*

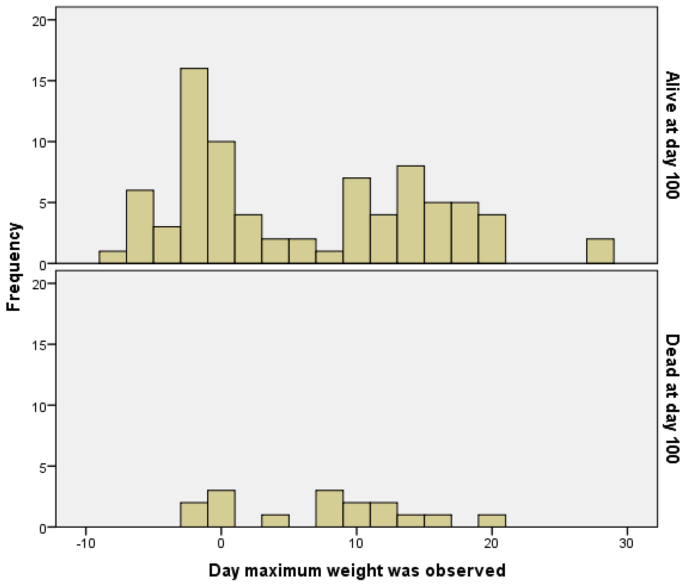

(**B**)

Figure S2. Early posttransplant weight gain in allotransplant recipients. The figures show the day of maximal weight gain after initiation of the conditioning treatment. Day 0 is the day of stem cell infusion. (**A**) This figure shows the day of maximal weight gain for all allotransplant recipients (n = 100); (**B**) The two figures show the day of maximal weight gain for patients being alive on day +100 posttransplant (**upper**) and for the patients being dead at this time (**lower**).
